# Targeting Wnt pathway in mantle cell lymphoma-initiating cells

**DOI:** 10.1186/s13045-015-0161-1

**Published:** 2015-06-06

**Authors:** Rohit Mathur, Lalit Sehgal, Frank K. Braun, Zuzana Berkova, Jorge Romaguerra, Michael Wang, M. Alma Rodriguez, Luis Fayad, Sattva S. Neelapu, Felipe Samaniego

**Affiliations:** Department of Lymphoma and Myeloma, The University of Texas MD Anderson Cancer Center, 1515 Holcombe Blvd., Houston, TX 77030 USA

**Keywords:** Lymphoma-initiating cells, Tumor stem cells, Burton tyrosine kinase, Wnt3, FZD1, Mesenchymal stromal cells, MCL co-culture, CCT036477, iCRT14, PKF118-310

## Abstract

**Background:**

Mantle cell lymphoma (MCL) is an aggressive and incurable form of non-Hodgkin’s lymphoma. Despite initial intense chemotherapy, up to 50 % of cases of MCL relapse often in a chemoresistant form. We hypothesized that the recently identified MCL-initiating cells (MCL-ICs) are the main reason for relapse and chemoresistance of MCL. Cancer stem cell-related pathways such as Wnt could be responsible for their maintenance and survival.

**Methods:**

We isolated MCL-ICs from primary MCL cells on the basis of a defined marker expression pattern (CD34-CD3-CD45+CD19-) and investigated Wnt pathway expression. We also tested the potential of Wnt pathway inhibitors in elimination of MCL-ICs.

**Results:**

We showed that MCL-ICs are resistant to genotoxic agents vincristine, doxorubicin, and the newly approved Burton tyrosine kinase (BTK) inhibitor ibrutinib. We confirmed the differential up-regulation of Wnt pathway in MCL-ICs. Indeed, MCL-ICs were particularly sensitive to Wnt pathway inhibitors. Targeting β-catenin-TCF4 interaction with CCT036477, iCRT14, or PKF118-310 preferentially eliminated the MCL-ICs.

**Conclusions:**

Our results suggest that Wnt signaling is critical for the maintenance and survival of MCL-ICs, and effective MCL therapy should aim to eliminate MCL-ICs through Wnt signaling inhibitors.

**Electronic supplementary material:**

The online version of this article (doi:10.1186/s13045-015-0161-1) contains supplementary material, which is available to authorized users.

## Background

Mantle cell lymphoma (MCL) is considered as an incurable subtype of non-Hodgkin’s lymphoma that causes significant morbidity and early death presumably due to relapsed disease [1–3]. Despite apparent clinical remissions achieved with chemotherapy regimens (R-CHOP or R-hyperCVAD), MCL relapse rates hover around 50 % [4, 5]. The relapse is considered to be due to chemoresistant cells that prevent complete elimination of MCL cells.

A small fraction of cells within tumors have tumor-initiating properties and are believed to be the source of relapsed cancer. These cells are referred to as cancer stem cells (CSCs) or tumor-initiating cells [6–9]. CSCs have been implicated in the growth, progression, and relapse of several tumor subtypes. The most current therapies target dividing tumor cells while sparing non-dividing and inherently chemoresistant CSCs; thus, they fail to provide long-term cures and result in tumor relapse [10, 11].

CSCs and normal hematopoietic stem cells share Wnt, Notch, and Hedgehog signaling pathways, which are required for their growth and self-renewal [7]. Recent studies have suggested a role of Wnt signaling in MCL tumorigenesis [12–14]. The Wnt signaling pathway regulates development, and its dysregulation leads to oncogenesis [15–17]. Canonical Wnt signaling is initiated by the binding of Wnt ligands to their cognate Frizzled (FZD) receptors and its co-receptors, low density lipoprotein receptor related proteins 5/6 (LRP5/6). In the absence of Wnt signaling, β-catenin is phosphorylated and its interaction with GSK-3β and axin-1 leads to its ubiquitination and degradation [18]. Activation of the Wnt pathway prevents β-catenin phosphorylation-induced degradation, and stabilized β-catenin accumulates in the nucleus, where it forms active transcription complexes with the T cell factor/lymphoid enhancer binding factor (TCF/LEF) family of DNA-binding transcription factors [19–21]. Dysregulation of Wnt pathway can promote tumorigenesis [22, 23]. Selective targeting of stem cell signaling pathways should eliminate CSCs [24].

MCL-initiating cells (MCL-ICs) have been recently identified based on a lack of CD19 marker (CD34-CD3-CD45+CD19- cells) [25]. Two studies from different groups have shown that these MCL-ICs can repopulate tumor in mice [25, 26]. As few as 100 of CD19- MCL-ICs have been found to produce whole tumor with both CD19+ and CD19- cells, while CD19+ MCL-non-ICs were incapable of tumor development at comparable limited dilutions in severe combined immunodeficiency (SCID) mice [25, 26]. We suggest that the high relapse rates of human MCL arise from incomplete elimination of chemoresistant MCL-ICs [27]. Thus, in order to improve long-term survival of individuals with MCL, it is important to have a fuller understanding of the signaling pathways responsible for the chemoresistance and maintenance of MCL-ICs. In this study, we investigated the expression and importance of Wnt pathway in survival of MCL-ICs and explored ways to eliminate these cells.

## Results

### MCL-ICs possess stem cell-like properties

**Fig. 1:**
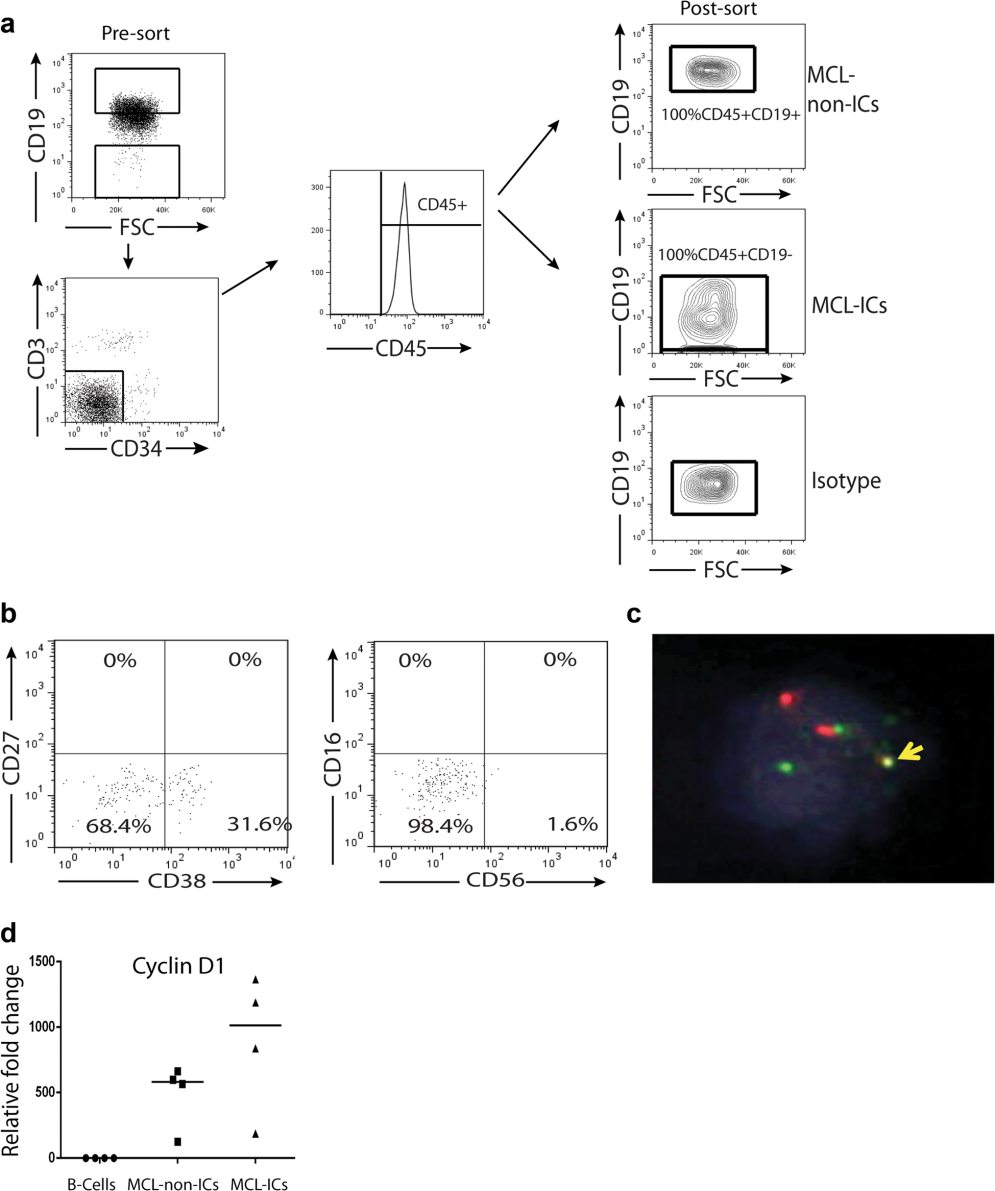
Isolation of MCL-ICs. (**a**) Isolation of MCL-ICs using immunostaining and flow sorting. (**b**) Immunostaining of isolated MCL-ICs for plasma cell markers CD27/CD38 and natural killer cell markers CD56/CD16 detected by flow cytometry. (**c**) Detection of gene fusion t (11;14) (q13; q32) in MCL-ICs using fluorescent in situ hybridization, indicated by *arrow*. (**d**) qRT-PCR expression of cyclin D1 in MCL-ICs, MCL-non-ICs relative to B-cells. Differences between MCL-ICs and B-cells were significant (*P* < 0.05) for cyclin D1

Subpopulations of MCL-ICs (CD34-CD3-CD45+CD19-) and MCL-non-ICs (CD34-CD3-CD45+CD19+) were isolated from a MCL tumor sample based on a previously described immunostaining and sorting protocol (Fig. 1a) [25]. The purity and identity of the isolated MCL-ICs population was confirmed by a lack of expression of surface markers for plasma cells (CD27, CD38) and natural killer cells (CD56, CD16) (Fig. 1b). Fluorescence in situ hybridization analysis of isolated MCL-ICs and cyclin D1 expression confirmed the presence of t (11;14) (q13; q32) (Fig. 1c). Presence of cyclin D1 overexpression in MCL-ICs confirmed that MCL-ICs are clonal cells (Fig. 1d).

**Fig. 2:**
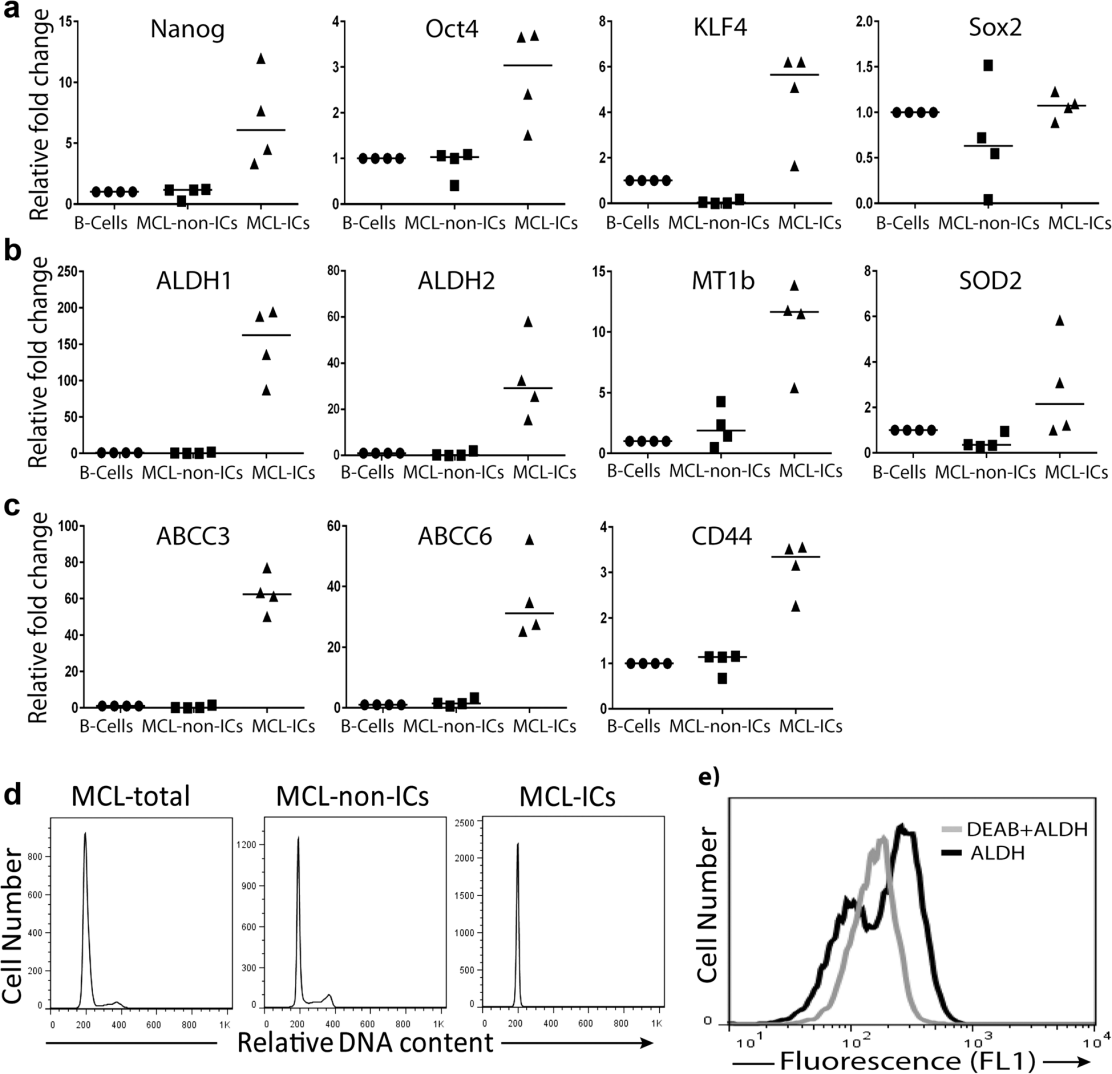
Stem cell-like properties of MCL-ICs. **a**–**c** qRT-PCR performed using the total cellular RNA isolated from MCL-ICs (*n* = 4) for **a** stem cell transcription factors (Nanog, Oct4, Sox2, Klf4), **b** ALDH isoforms and antioxidant enzymes SOD2 and MT1b, and **c** chemoresistance-associated genes encoding ABCC3, ABCC6, and CD44. Differences between MCL-ICs and MCL-non-ICs were significant (*P* < 0.05) for ALDH1, ALDH2, SOD2, MT1b, Nanog, Oct4, Klf4, ABCC3, ABCC6, and CD44. **d** Cell cycle analysis of isolated MCL-ICs, MCL-non-ICs, and total MCL cells by flow cytometry. **e** ALDH activity in freshly isolated MCL-ICs from apheresis samples evaluated using ALDEFLUOR kit

qRT-PCR analysis revealed enrichment of the stem cell core transcription factors Nanog, Oct4, and KLF4 (5.29, 3.06, and >100-fold, respectively) in MCL-ICs compared with MCL-non-ICs (Fig. 2a). However, Sox2 expression was not significantly elevated in MCL-ICs (1.07-fold) compared with B-cells (peripheral blood CD19+ cells). qRT-PCR analysis also showed significantly higher (>100-fold) expression of aldehyde dehydrogenase 1 (ALDH1) and ALDH2 in MCL-ICs than in MCL-non-ICs (Fig. 2b); this observation concurs with the high ALDH activity detected in MCL-ICs (Fig. 2e). The expression levels of the antioxidant enzymes MT1b and SOD2 were elevated over sixfold in MCL-ICs, suggesting a higher reactive oxygen species scavenging capacity (Fig. 2b). MCL-ICs also overexpressed genes associated with chemoresistance, such as those encoding the ATP transporters ABCC3 and ABCC6 as well as CD44 (>100-, 22-, and 3-fold, respectively) compared with MCL-non-ICs (Fig. 2c). Cell cycle analysis showed that 100 % of MCL-ICs were quiescent (in G0/G1 phase), whereas MCL-non-ICs were distributed throughout all phases of the cell cycle (G0/G1, 69.2 %; S, 9.16 %; G2/M, 15.5 %) (Fig. 2d). Taken together, these results indicate that MCL-ICs possess characteristic gene expression of cancer stem cells.

### Wnt pathway genes are overexpressed in MCL-ICs

**Fig. 3:**
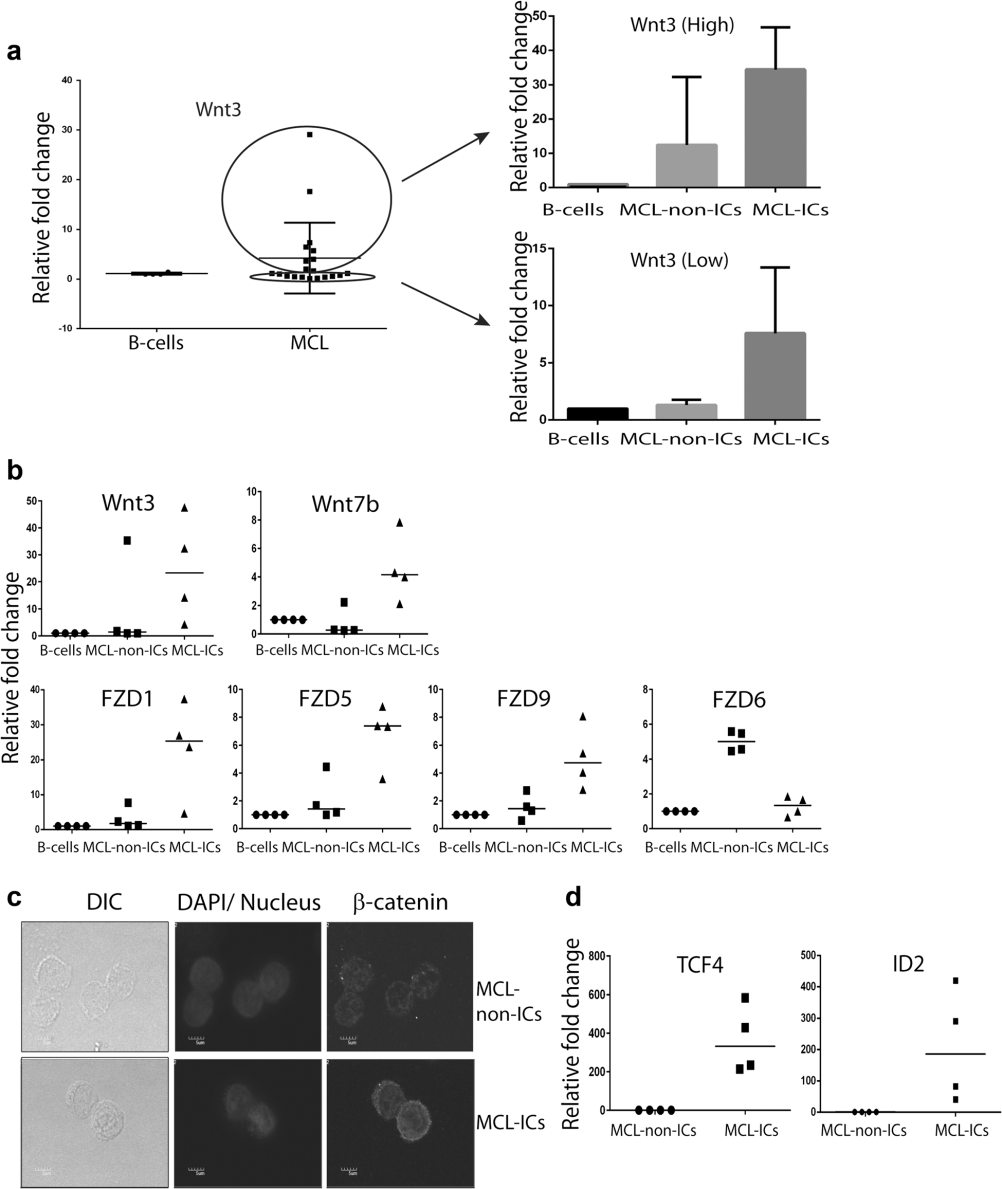
Enrichment of Wnt signaling pathway genes in MCL-ICs. **a** Expression of Wnt3 in unfractionated MCLs (*n* = 20) and MCL-ICs isolated from unfractionated MCLs expressing high (*n* = 3) and low (*n* = 3) Wnt3. **b** Expression of mRNAs encoding Wnt ligands and FZD receptors in freshly isolated MCL-ICs and MCL-non-ICs relative to B-cells from healthy donors. Horizontal lines represent median for each group. Differences between MCL-ICs and MCL-non-ICs were significant (*P* < 0.05) for Wnt3, Wnt7b, FZD1, FZD5, FZD9, and FZD6. **c** Immunostaining detection of the expression and localization of β-catenin in freshly isolated MCL-ICs and MCL-non-ICs. Color image is included in Additional file 1: Figure S1. **d** Relative expression levels of Wnt target genes encoding ID2 and β-catenin–interacting transcriptional factor TCF4 in MCL-ICs (*n* = 4) and MCL-non-ICs (*n* = 4). Differences between MCL-ICs and MCL-non-ICs were significant (*P* < 0.05) for both genes

Analysis from previous studies using unfractionated MCL cells have implicated the Wnt pathway in the pathogenesis of mantle cell lymphoma [12–14]. Therefore, we first investigated Wnt3 expression in unfractionated MCL. Our observations suggest that 9 out of 20, nearly 45 % MCL samples, overexpress Wnt3. We next investigated the expression of Wnt3 in MCL-ICs isolated from MCL samples expressing high and low Wnt3 levels. Our results showed that MCL-ICs were enriched in Wnt3 compared to MCL-non-ICs and B-cells, irrespective of total tumor Wnt3 expression (Fig. 3a). We observed differential up-regulation of Wnt ligands and their FZD receptors in MCL-ICs compared with MCL-non-ICs (Fig. 3b, Table 1), using B-cells as a reference. To show other evidence of enhanced Wnt signaling, we performed immunostaining for β-catenin. Higher cellular and nuclear levels of β-catenin were observed in MCL-ICs than in MCL-non-ICs (Fig. 3c, Additional file 1: Figure S1) whereas B-cells did not show detectable β-catenin levels (Additional file 1: Figure S1). Activation of Wnt signaling in MCL-ICs was confirmed by the elevated expression of the Wnt target genes encoding ID2 and TCF4 (both >100-fold) compared with MCL-non-ICs (Fig. 3d). Thus, by 3 independent methods, we show that the Wnt pathway is differentially up-regulated in MCL-ICs.

**Table 1:** qRT-PCR analysis of Wnt ligands and FZD receptor expression in primary MCL cells compared to B-cells from healthy donors

	MCL-non-ICs		MCL-ICs			*P* value
	Median	95 % CI		Median	95 % CI	
Wnt Ligands						
Up-regulated in MCL-non-ICs and MCL-ICs						
Wnt3a	17.555	0.12–56.05		34.52	2.49–91.86	0.0877
Wnt11	3.66	0.19–8.91		32.61	0.84–69.97	0.1588
Up-regulated in MCL-ICs						
Wnt5a	1.78	0.76–21.93		10.5	3.56–17.87	0.3465
Wnt3	1.49	0.98–35.36		23.32	4.26–47.61	0.0500
Wnt8b	1.25	0.39–2.60		3.89	1.45–15.16	0.2308
Wnt4	1.76	0.21–6.13		3.41	0.54–5.65	0.2820
Wnt7a	1.21	0.75–5.32		2.09	0.60–6.76	0.2715
Wnt6	1.02	0.05–17.65		1.85	0.08–8.71	0.5133
Wnt5b	0.66	0.42–18.82		45.47	0.95–146.30	0.1956
Wnt1	0.45	0.15–12.25		9.26	4.18–15.20	0.1528
Wnt7b	0.28	0.25–2.24		4.15	2.13–7.85	0.0156
Wnt9b	0.39	0.15–1.47		2.44	1.08–8.79	0.1764
Wnt2b	0.28	0.16–8.18		1.39	0.05–9.60	0.0802
Wnt10a	0.67	0.24–14.80		0.96	0.03–4.41	0.5622
Up-regulated in MCL-non-ICs						
Wnt9a	229.93	4.51–962.28		18.31	10.81–304.06	0.3201
Wnt16	11.99	1.32–32.97		4.61	3.23–9.86	0.3029
Wnt8a	6.97	0.24–18.73		1.01	0.15–2.32	0.1785
FZD Receptors						
Up-regulated in MCL-non-ICs and MCL-ICs						
Fz2	37.31	4.24–58.26		46.96	10.01–157.60	0.2909
Fz7	4.53	0.49–9.76		4.48	1.89–9.04	0.9536
Up-regulated in MCL-ICs						
Fz4	1.15	0.07–2.52		122.7	1.93–1340	0.3002
Fz1	1.8	1.10–7.76		25.41	4.65–37.39	0.0390
Fz5	1.43	0.99–4.45		7.37	3.58–8.77	0.0100
Fz9	1.44	0.59–2.75		4.74	2.81–8.08	0.0379
Fz10	0.64	0.29–1.44		4.26	3.91–10.99	0.0738
Fz8	0.13	0.01–0.39		0.77	0.10–4.76	0.2907
Up-regulated in MCL-non-ICs						
Fz6	5.02	4.47–5.59		1.34	0.66–1.85	0.0001
Fz3	0.3	0.12–7.04		0.26	0.24–1.22	0.4814

B-cells (median = 1) are used as reference. Median with 95 % confidence interval limits depicts the variations observed among patient samples. Differences between MCL-ICs and MCL-non-ICs were considered as significant with *P* < 0.05.

*MCL* mantle cell lymphoma, *ICs* initiating cells

### Inhibition of Wnt signaling preferentially eliminates MCL-ICs

**Fig. 4:**
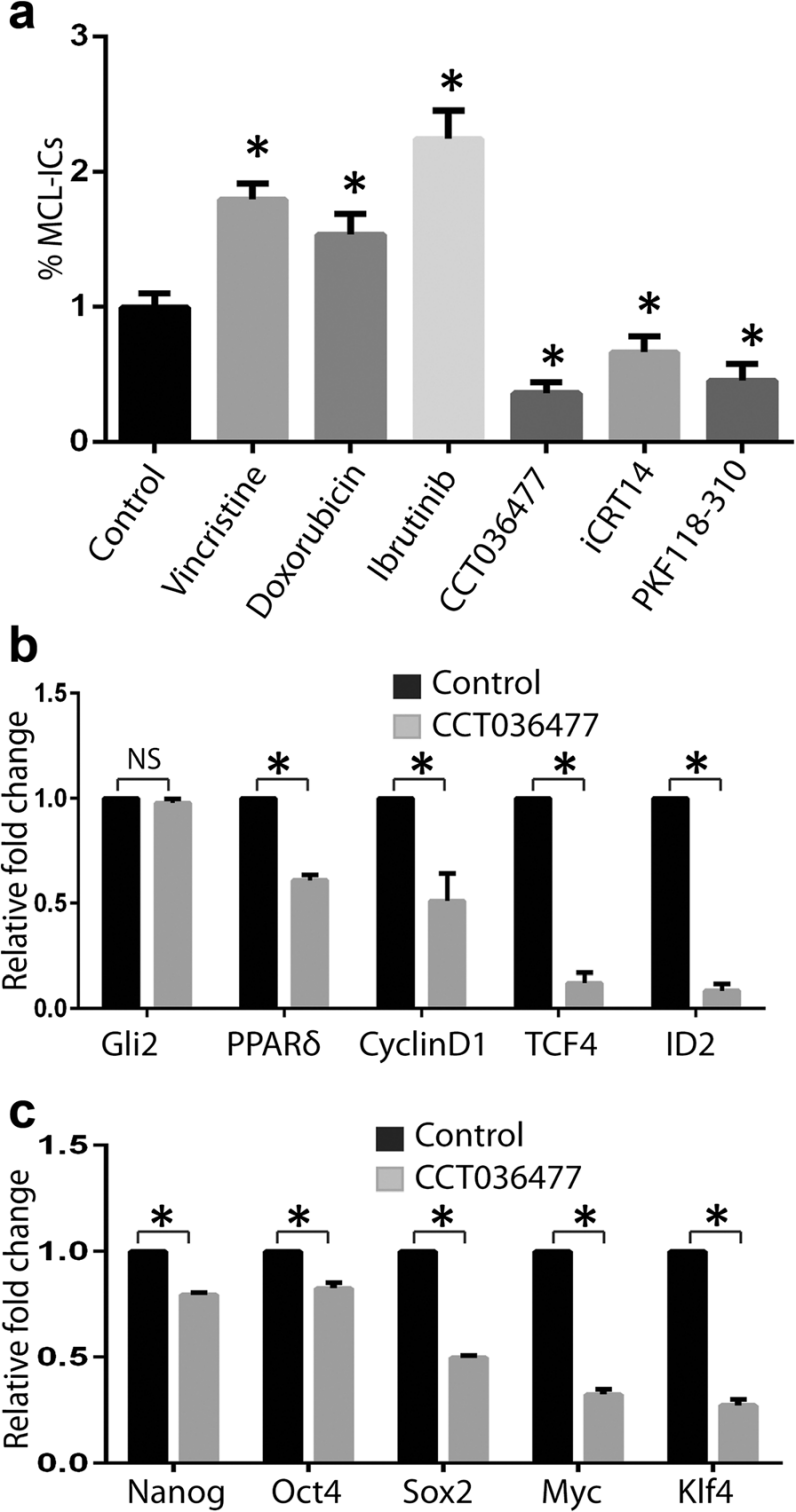
Preferential elimination of MCL-ICs by inhibition of Wnt signaling. **a** Percentage of MCL-ICs evaluated by immunostaining and flow cytometry (as shown in Fig. 1a) of primary MCL cells (*n* = 3) treated with vincristine (5 nM), doxorubicin (35 nM), or ibrutinib (10 μM), the Wnt inhibitors, CCT036477 (10 μM), iCRT14 (10 μM), or PKF118-310 (10 μM), for 48 h. *Differences between treated and control group were significant *P* < 0.05 (**b–c**) qRT-PCR analysis of the expression of (**b**) Wnt target genes encoding PPARδ, Cyclin D1, Myc, TCF4, ID2, and (**c**) stem cell core transcription factors Nanog, Oct4, Myc, Sox2, and Klf4 in MCL-ICs (*n* = 3) treated with 10 μM CCT036477 for 6 h. Gli2 is an off-target control. *P < 0.05

**Fig. 5:**
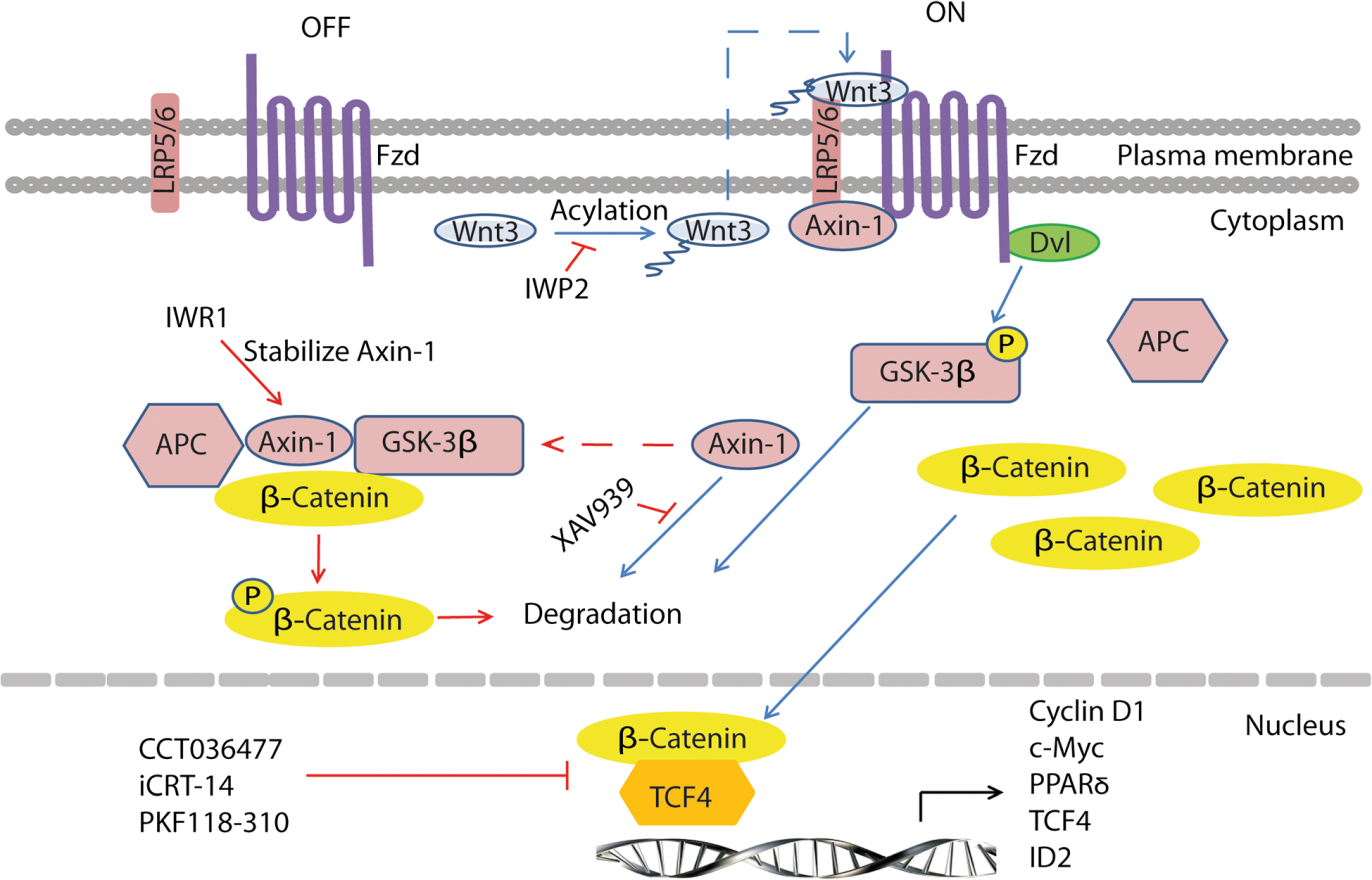
Schematic representation of Wnt pathway inhibition by small molecule inhibitors. Wnt pathway is turned off in the absence of Wnt ligand (left); destruction complex involving APC, Axin-1, and GSK-3β interacts with and phosphorylates β-catenin leading to its degradation. Binding of Wnt ligands to their cognate Frizzled (FZD) receptors and its co-receptors, low density lipoprotein receptor related proteins 5/6 (LRP5/6), activates the Wnt pathway (right) leading to sequestration and degradation of Axin-1 and phosphorylation and degradation of GSK-3β. Degradation of destruction complex components leads to accumulation of β-catenin and its subsequent translocation into the nucleus where it interacts with TCF4 to promote transcription of Wnt target genes. IWR1 stabilizes Axin-1 and promotes formation of destruction complex and degradation of β-catenin (*red arrows*). IWP2 inhibits Porcupine-mediated acylation and subsequent secretion of Wnt ligands. XAV939 inhibits Tankyrase-mediated degradation of Axin-1 and thus promotes formation of destruction complex (*red dashed arrow*). CCT036477, iCRT14, and PKF118-310 target β-catenin-TCF4 transcription complex

Treatment of primary MCL cells with chemotherapeutic drugs (vincristine, doxorubicin, or ibrutinib) induced apoptosis in MCL cells but did not decrease the percentage of MCL-ICs (1.79, 1.57, and 2.18 %, respectively) compared with buffer control (1 % MCL-ICs) suggesting chemoresistance of MCL-ICs to these agents (Fig. 4a). We analyzed the effects of Wnt signaling inhibitors targeting the pathway either upstream of β-catenin degradation (tankyrase inhibitor XAV939, axin-1 stabilizer IWR1-endo, and porcupine inhibitor IWP2) or downstream at β-catenin-mediated transcription complex (CCT036477, iCRT14, and PKF118-310) (Fig. 5). MCL cells were treated with the known active concentrations of these inhibitors and evaluated for the percentage of MCL-ICs. None of the agents acting upstream of β-catenin degradation decreased the percentage of MCL-ICs. On the other hand, chemical inhibitors of β-catenin-TCF4 interaction, CCT036477, iCRT14, and PKF118-310, effectively decreased the percentage of MCL-ICs from 1 % in buffer control to 0.35, 0.68, and 0.44 %, respectively (Fig. 4a) and induced apoptosis of MCL cells (Additional file 2: Figure S2). We next examined the effect of the most potent Wnt inhibitor, CCT036477, on the expression of Wnt target genes and transcription factors associated with stemness of MCL-ICs. Treatment with CCT036477 reduced the expression of the Wnt target genes encoding PPARδ, Cyclin D1, TCF4, and ID2 (1.64-, 1.96-, 2.56-, 8.33-, and 12.5-fold, respectively) (Fig. 4b), and the stem cell-specific core transcription factors Nanog, Oct4, Sox2, Myc, and Klf4 (1.28-, 1.26-, 2-, 3.26-, and 3.67-fold, respectively) (Fig. 4c). Gli2 was used as off-target negative control. In contrast, inhibitors of Hedgehog and Notch signaling pathways did not decrease the percentage of MCL-ICs (Additional file 3: Figure S3). Taken together, these results suggest that targeting β-catenin-TCF4 interaction can preferentially eliminate MCL-ICs by effectively blocking Wnt signaling in MCL-ICs.

## Discussion

The high rate of MCL relapse after initial apparent clinical remissions achieved with conventional chemotherapy suggests incomplete elimination of MCL cells and implicates a role for chemoresistant MCL-ICs in relapse. Here we showed that MCL-ICs have functional properties of cancer stem cells: high expression of ALDH, antioxidant enzymes, chemoresistance-associated genes, and stem cell-associated transcription factors, while still retaining t (11;14) (q13; q32) and overexpression of cyclin D1. Our analysis showed that MCL-ICs overexpress a subset of Wnt ligands and FZD receptors and that Wnt signaling is activated in MCL-ICs. Treatment of primary MCL cells with Wnt inhibitors preferentially eliminated MCL-ICs, which was not achieved with the current chemotherapy agents vincristine, doxorubicin, or even with the recently FDA-approved agent ibrutinib [28]. Burton tyrosine kinase (BTK) has been shown to be a negative regulator of Wnt signaling [29]. Therefore, it is not surprising that ibrutinib (a BTK inhibitor) probably resulted in inducing Wnt signaling rather than inhibiting it and thereby could not eliminate MCL-ICs. Our results suggest that the inability of conventional chemotherapy to kill MCL-ICs can be overcome by adding inhibitors of Wnt signaling.

A recent study showed that cobble stone area-forming cells (CAFCs) that developed from MCL co-cultured with human mesenchymal stem cells (hMSCs) are the morphologic equivalent of MCL-ICs with the CD34-CD3-CD45+CD19-CD133+ marker phenotype and manifested their tumor-initiating capacity in NOD/SCID mice [26]. CAFCs were also resistant to bortezomib, fludarabine, and doxorubicin and expressed stem cell transcription factors Nanog and Oct4 but not Sox2 [26]. The CD34-CD3-CD45+CD19- MCL-ICs characterized in our study are identical to the CAFCs; they were also CD133+ and exhibited the same characteristics. However, our study has further extended the characterization of MCL-IC, by identifying a hyperactive Wnt signaling pathway, crucial for their maintenance and survival.

Our results showed up-regulated expression of canonical ligand Wnt3 [30] but not of the non-canonical ligands such as Wnt4, Wnt5, and Wnt11 [31] in MCL-ICs compared to MCL-non-ICs. Immunostaining results also confirmed the differentially higher staining of active unphosphorylated β-catenin in MCL-ICs, which is required for canonical but not for non-canonical Wnt pathway [32]. In addition, the FZD6 receptor, which is associated with inhibition of canonical Wnt signaling pathway [33], was not differentially expressed in MCL-ICs. These results clearly indicate the presence of activated canonical Wnt signaling pathway in MCL-ICs.

Other investigators have revealed that Wnt is an important pathway in primary MCL tissues and have implicated this pathway in the pathogenesis of MCL [13]. Analysis of unfractionated MCL shows only a threefold up-regulation compared to B-cells in the previous study [13]. Our study showed a 23-fold enhanced Wnt3 expression in MCL-ICs compared to B-cells. These results clearly show the importance of Wnt signaling in MCL-ICs.

MCL is believed to be driven by enhanced cyclin D1 expression due to t (11;14) (q13; q32) present in >90 % of MCL [34, 35]. A minority of MCLs do not express cyclin D1 [36]. However, other isoforms of cyclin D are overexpressed in cyclin D1-negative MCLs, which suggests an indispensable requirement for the expression of at least one isoform of cyclin D in MCL [37]. Thus, it appears that mechanisms other than t (11;14) (q13; q32) are responsible for the overexpression of at least one other cyclin D in cyclin D1-negative MCL. It is of interest that Wnt signaling could potentially fulfill this role [38–40] as it was shown to up-regulate cyclin D2 expression [41, 42]. Wnt3 was also noted to be overexpressed in cyclin D1-negative MCL that also lacked t (11;14) (q13; q32) [36]. Therefore, enhanced Wnt signaling may be the underlying driver of overexpression of cyclin D in all MCL regardless of t (11;14) status.

Overexpression of Wnt ligands and their cognate FZD receptors in MCL-ICs points to the existence of an autocrine signaling loop. Immunostaining of β-catenin and the elevated expression of Wnt target genes encoding ID2 and TCF4 clearly confirmed higher Wnt activity in MCL-ICs than in MCL-non-ICs. However, inhibitors of Wnt signaling acting upstream of β-catenin degradation step had no effect on MCL-ICs. On the other hand, downstream Wnt inhibitors targeting the β-catenin-TCF4 transcription complex (CCT036477, iCRT14, and PKF118-310) preferentially eliminated MCL-ICs over MCL-non-ICs. These findings can be explained either by the very low expression of proteins involved in β-catenin degradation such as GSK-3β and axin-1 (target of XAV939, IWR1-endo, IWP2 compounds) observed in MCL-ICs or by a contribution of redundant and additional pathways to β-catenin activation, such as autocrine fibroblast growth factor receptor signaling [43, 44]. Further experiments will be required to delineate the major contributors to β-catenin activation in MCL-ICs.

We found elevated levels of FZD1 and its ligand Wnt3 in MCL-ICs as compared to MCL-non-ICs. Other researchers have demonstrated that targeting FZD1 reverses multidrug resistance in neuroblastomas and breast cancer cells [45, 46]. Thus, Wnt3-FZD1 signaling may be one of the reasons for chemoresistance in MCL [47, 48].

In summary, we have outlined an enhanced Wnt/β-catenin signaling in MCL-ICs and shown that inhibition of the Wnt pathway effectively eliminates MCL-ICs, which are implicated in MCL relapse. We have also demonstrated that present therapy approaches for MCL, including recently approved drug ibrutinib, do not address the killing of MCL-ICs. Thus, we anticipate that current rates of MCL relapse will not decrease substantially with current therapies. A detailed examination of selectively enhanced signaling in MCL-ICs may be a good starting point to expose pathways important for MCL tumor stem cell survival. Presenting clinical and MCL features at time of initial MCL lymphoma presentation do not identify a priori, who are the patients who will relapse from those will attain cures. Perhaps, studying the MCL-IC and their response to targeting agents will be a key to identify patients who will relapse. Our results clearly show that Wnt signaling inhibitors targeting β-catenin-TCF4 interaction can eliminate MCL-ICs. However, blocking the Wnt pathway exclusively in tumor cells will be challenging, as Wnt signaling also has a role in the self-renewal of non-malignant tissues such as intestinal crypts, and bone growth plates [49, 50]. Nevertheless, our results point to the important and actionable targets of Wnt signaling in MCL pathogenesis and its potential usefulness as a target for therapy to eliminate MCL-ICs and reduce the risk of MCL relapse.

## Conclusions

Our results clearly demonstrate the differential activation of Wnt pathway in MCL-ICs. Not all steps in the Wnt signaling pathway are amenable to effective blocking in MCL. We show that blocking of Wnt signaling at the β-catenin-TCF4 transcription complex effectively blocks signaling in MCL-IC and preferentially kills the MCL-IC cells, which harbor chemoresistance. This study results shows identification of effective agents in MCL-IC that would not had been possible by studying whole MCL cells. Since inhibition of Wnt pathway resulted in preferential elimination of MCL-ICs, we conclude that Wnt pathway should be targeted to eliminate MCL-ICs and reduce the risk of relapsed MCL.

## Methods

### Patients and agents

Cells and clinical information from MCL patients described in this manuscript (Additional file 4: Table S2) were collected and published with the written informed consent of each patient under The University of Texas MD Anderson Cancer Center IRB-approved clinical protocol LAB08-0190 for use of human tissues.

The following agents were tested: Wnt inhibitors XAV939 (Selleck Chemicals, Houston, TX), iCRT14 (R&D Systems, Minneapolis, MN), CCT036477, PKF118-310 (Sigma-Aldrich, St. Louis, MO), IWP2, IWR1-endo, and IWR1-exo (Santa Cruz Biotechnologies, Santa Cruz, CA); Hedgehog inhibitors GANT61 (R&D Systems, Minneapolis, MN), LDE225 and Cyclopamine (both from Selleck Chemicals, Houston, TX); Notch inhibitor RO4929097 (Selleck Chemicals, Houston, TX).

### Isolation of normal B-cells

Peripheral blood B-cells were isolated from healthy donors’ blood obtained from the Gulf Coast Blood Center (Houston, TX) by using CD19-positive magnetic beads and were released with the competitive CD19 DETACHaBEAD according to the manufacturer’s instructions (Invitrogen-Life Technologies, San Diego, CA). All procedures were performed under The University of Texas MD Anderson Cancer Center IRB-approved clinical protocol LAB08-0190.

### Isolation of MCL cells and MCL-ICs

MCL tumor cell-enriched buffy coats were isolated from apheresis or leukemic phase blood of MCL patients by Histopaque-1077 (Sigma-Aldrich, St. Louis, MO) gradient centrifugation. Obtained cells were then stained with antibodies against CD34-APC (Cat No. 555824), CD3-APC-Cy7 (Cat No. 557832), CD45-FITC (Cat No. 555482), CD19-PE (Cat No. 555413), and Sytox blue for selection of live cells (all from BD Bioscience, San Jose, CA). Subpopulations of MCL-ICs (CD34-CD3-CD45+CD19-) and MCL-non-ICs (CD34-CD3-CD45+CD19+) were isolated using a fluorescence-activated cell sorter (Influx, BD Bioscience, San Jose, CA) according to a previously described protocol [25]. Subpopulations of sorted cells were analyzed for purity by immunostaining with markers for plasma cells (CD27, CD38) and natural killer cells (CD56, CD16) using the antibodies CD27-PerCP-Cy5.5 (Cat No. 560612), CD38-PE-Cy7 (Cat No. 560677), CD56-PE-Cy7 (Cat No. 557747), and CD16-Pacific blue (Cat No. 558122) (all from BD Bioscience, San Jose, CA), respectively.

### ALDH activity assay

ALDH activity in cells was determined by using an ALDEFLUOR kit according to the manufacturer’s protocol (STEMCELL Technologies, Vancouver, Canada). Briefly, 1 × 10^6^ cells were resuspended in a 1 ml assay buffer with 5 μl of ALDEFLOUR reagent. DEAB was used as inhibitor of ALDH activity. A 500-μl aliquot of the reagent mixed cells was transferred to an Eppendorf tube containing 5 μl of DEAB as a control. Samples were incubated at 37 °C for 45 min. Green fluorescence intensity was measured with a BD Fortessa flow cytometer (Becton Dickinson, San Jose, CA) and evaluated with FlowJo software (Tree Star, Ashland, OR).

### Fluorescent in situ hybridization

Isolated MCL-ICs, MCL-non-ICs, and B-cells from healthy donors were affixed on slides (Statlab, McKinney, TX) using cytospin. Slides were fixed using SAFETEX cytology spray (Andwin Scientific, Woodland Hills, CA) and hybridized using Vysis IgH/CCND1 probe kit (Abbot molecular, Abbott park, IL) to confirm the presence of t (11;14) (q13; q32). Staining was assessed using a Bioview Duet imaging system (Bioview, Nes Ziona, Israel) equipped with an Olympus BX61 microscope (Olympus America, Center Valley, PA).

### Quantitative real-time polymerase chain reaction (qRT-PCR)

Total RNA was extracted from cells using RNAqueous kit according to the manufacturer’s protocol (Ambion-Life Technologies, Austin, TX). First-strand cDNA was synthesized using a Superscript III reverse transcriptase kit according to the manufacturer’s protocol (Invitrogen-Life Technologies, San Diego, CA). Samples were analyzed on 96-well microtiter plates using the StepOnePlus real-time PCR System (Applied Biosystems, Grand Island, NY). qRT-PCR was performed using SYBR green dye and primers specific for selected human genes (Additional file 5: Table S1) as described earlier [51, 52]. PCR was performed with 40 cycles of 95 °C for 15 s and 60 °C for 1 min. Step-One software version 2.1 was used to analyze the qRT-PCR data.

### Immunostaining

Cells were immobilized on glass slides by using cytospin prior to fixation in methanol for 1 h at −20 °C. Cells were permeabilized using 0.5 % Triton-×100 in PBS for 20 min at room temperature prior to staining with non-phosphorylated (active) anti-β-catenin antibody (1 μg, Cat No. 8814S, Cell Signaling, Danvers, MA) and AlexaFluor-488-conjugated secondary antibody (1:500, Cat No. A11008, Life Technologies, San Diego, CA). Slides were washed with 0.1 % Tween 20 and mounted with ProLong Gold antifade reagent containing nuclear stain 4′,6 diamidino-2-phenylindole dihydrochloride (DAPI) (Invitrogen-Life Technologies, San Diego, CA). Images were acquired at 60× using A1R confocal laser microscope system (Nikon Instruments, Melville, NY).

### Growth and treatment of MCL cells

Primary MCL cells were seeded onto a layer of human bone marrow mesenchymal stromal cells (hMSCs) at a MCL to a stromal cell ratio of 10:1 and grown in hMSC medium supplemented with mesenchymal cell growth factors and glutamine (Lonza, Allendale, NJ) at 37 °C in 5 % CO_2_ as described previously [53]. MCL cells were harvested, resuspended in 50 % fresh and 50 % conditioned medium from hMSC cultures, and incubated with the indicated agents for 6–48 h. Cells were either stained with propidium iodide for cell cycle analysis as described earlier [54] or had RNA isolated for further analysis. The percentage of MCL-ICs was determined using procedures described in the “Isolation of MCL cells and MCL-ICs” section above.

### Statistical analysis

Experimental data are reported as means or medians with standard deviation or error of mean, unless otherwise indicated. Differences between groups were calculated using the two-tailed Student’s *t* test (GraphPad Prism, GraphPad Software, Inc, La Jolla, CA). *P* < 0.05 was considered statistically significant.

## Additional files

Additional file 1: Figure S1. Wnt signaling pathway is active in MCL-ICs. Detection β-catenin expression and localization by immunofluorescence and confocal microscopy in (a) B-cells from healthy donors and in (b) freshly isolated MCL-ICs, and MCL-non-ICs. Additional file 2: Figure S2. Inhibition of Wnt signaling induce apoptosis of primary MCL cells. (a) Percentage apoptosis, sub-G1 analysis of primary MCL cells (*n* = 3) treated with vincristine (5 nM), doxorubicin (35 nM), or ibrutinib (10 μM), the Wnt inhibitors, CCT036477 (10 μM), iCRT14 (10 μM), or PKF118-310 (10 μM), for 48 h. *Differences between treated and control group were significant *P* < 0.05. Additional file 3: Figure S3. Hedgehog and Notch signaling pathways in MCL-ICs. Expression of mRNA encoding (a) Hedgehog signaling pathway transcription factors Gli1, Gli2, Gli3 and (b) Notch signaling target genes Hes1, Hes5 and Hey1 in freshly isolated MCL-ICs, and MCL-non-ICs relative to B-cells from healthy donors. Horizontal lines represent median for each group. Differences between MCL-ICs and MCL-non-ICs were significant (*P* < 0.05) for Gli3, and Hey1. (c) Percentage of MCL-ICs evaluated by immunostaining and flow cytometry (as shown in Fig. 1a) of primary MCL cells (*n* = 3) treated with Hedgehog inhibitors LDE225 (5 μM), Cyclopamine (5 μM), GANT61 (5 μM) and Notch inhibitor RO4929097 (5 μM) for 48 h. *Differences between treated and control group were significant *P* < 0.05. Overexpression of Gli3, a repressor of hedgehog pathway [55, 56] in MCL-ICs and inability of hedgehog and notch signaling pathway inhibitors to decrease percentage of MCL-ICs, suggest that these pathways may not be effective targets for reducing the percentage of MCL-ICs. Additional file 4: Table S2. Clinical information of patients submitting primary MCL tissue for analysis. Additional file 5: Table S1. List of primers.

## Abbreviations

CSCs: Cancer stem cells
MCL: Mantle cell lymphoma
MCL-IC: Mantle cell lymphoma-initiating cells
R-CHOP: Rituximab, cyclophosphamide, hydroxydaunorubicin, oncovin, prednisone
R-hyperCVAD: Rituximab, cyclophosphamide, vincristine, adriamycin, dexamethasone
ALDH: Aldehyde dehydrogenase
SCID: Severe combined immunodeficiency
BTK: Burton tyrosine kinase
FZD: Frizzled
hMSC: human mesenchymal stem cells
GSK-3β: Glycogen synthase kinase
CAFC: Cobblestone area-forming cells
