# Supplementary figures and images for: Targeting Wnt pathway in mantle cell lymphoma-initiating cells
Source: J Hematol Oncol. 2015 Jun 6;8:63. doi: 10.1186/s13045-015-0161-1 (PMC4460883; doi:10.1186/s13045-015-0161-1)

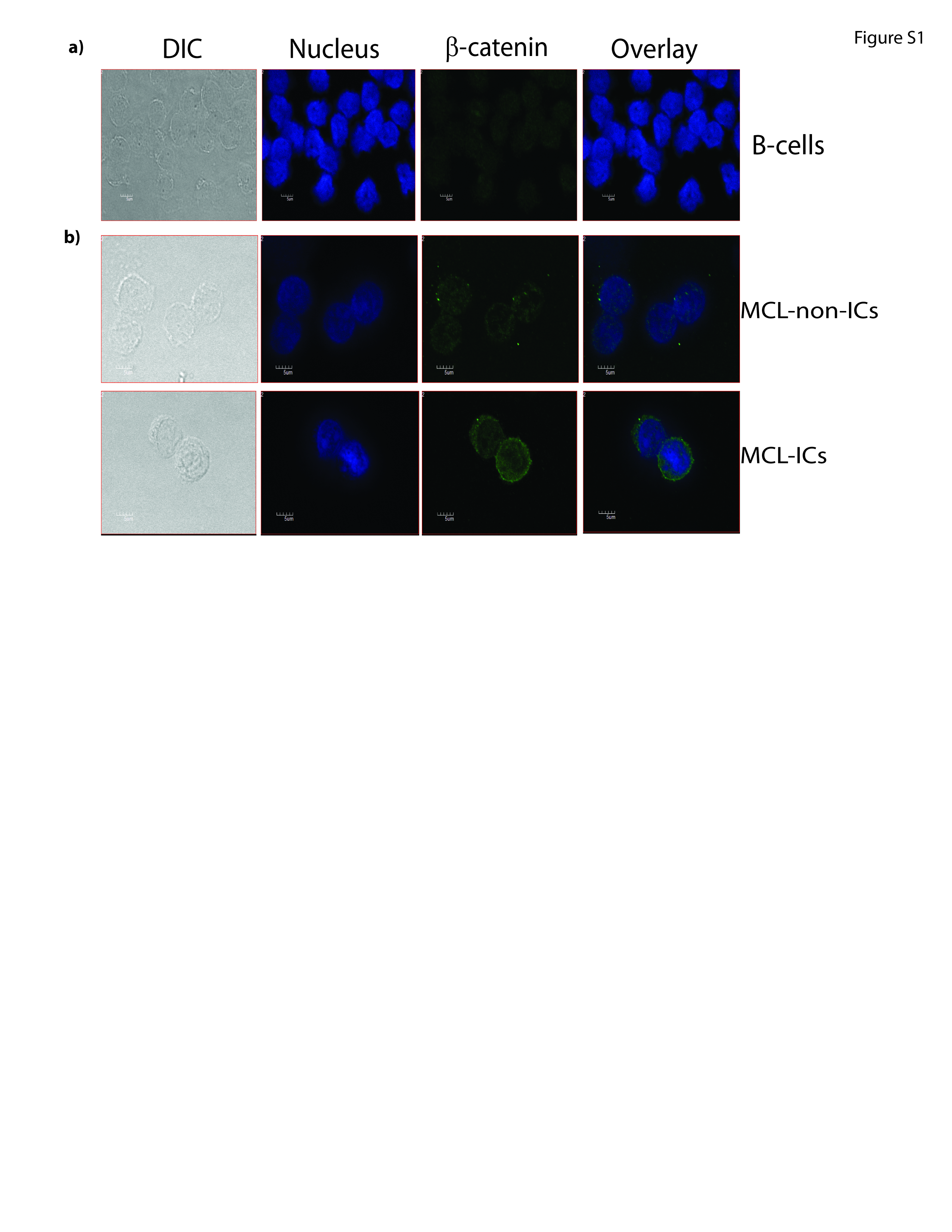

Supplement: Additional file 1: Figure S1. — Wnt signaling pathway is active in MCL-ICs. Detection β-catenin expression and localization by immunofluorescence and confocal microscopy in (a) B-cells from healthy donors and in (b) freshly isolated MCL-ICs, and MCL-non-ICs. [file 13045_2015_161_MOESM1_ESM.tif]

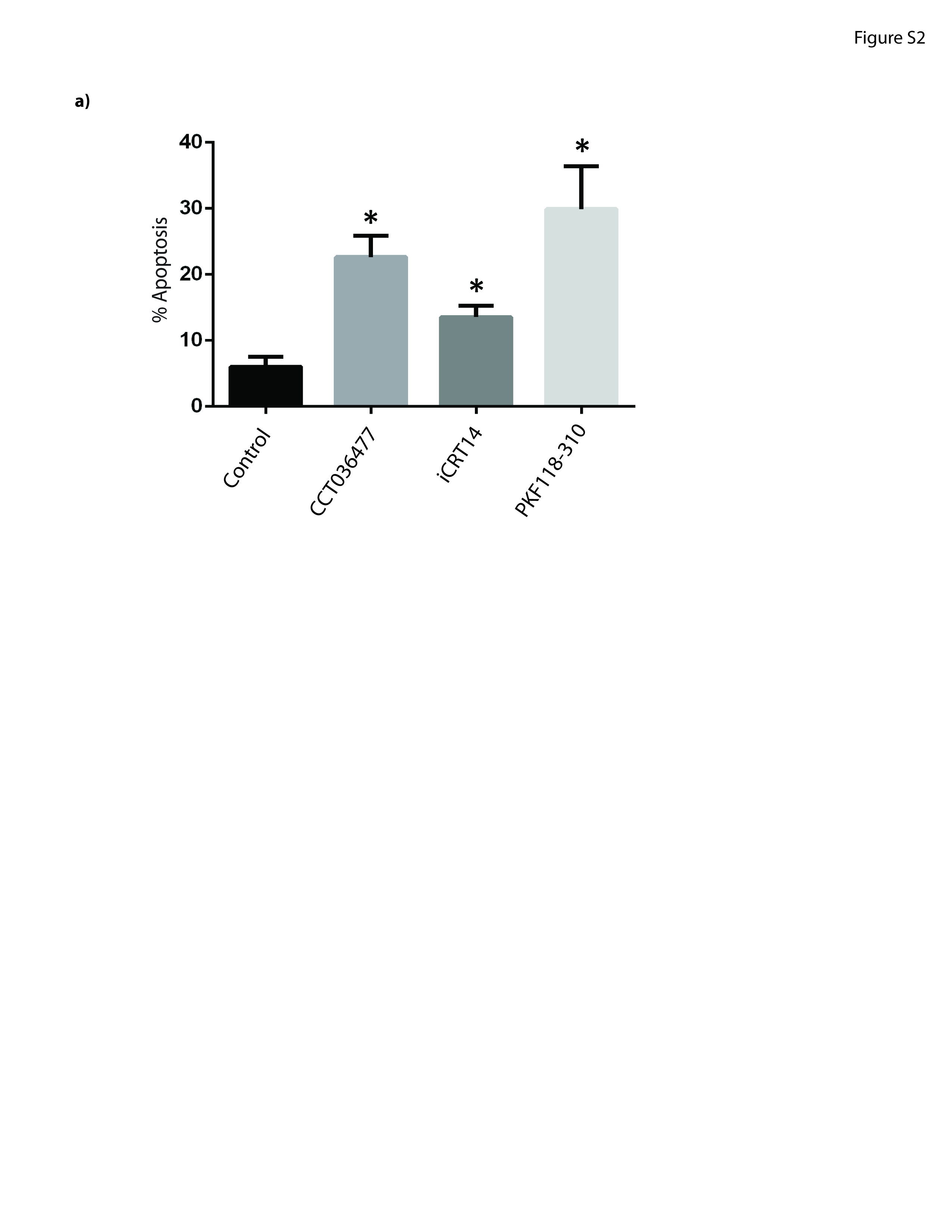

Supplement: Additional file 2: Figure S2. — Inhibition of Wnt signaling induce apoptosis of primary MCL cells. (a) Percentage apoptosis, sub-G1 analysis of primary MCL cells (n = 3) treated with vincristine (5 nM), doxorubicin (35 nM), or ibrutinib (10 μM), the Wnt inhibitors, CCT036477 (10 μM), iCRT14 (10 μM), or PKF118-310 (10 μM), for 48 h. *Differences between treated and control group were significant P < 0.05. [file 13045_2015_161_MOESM2_ESM.tif]

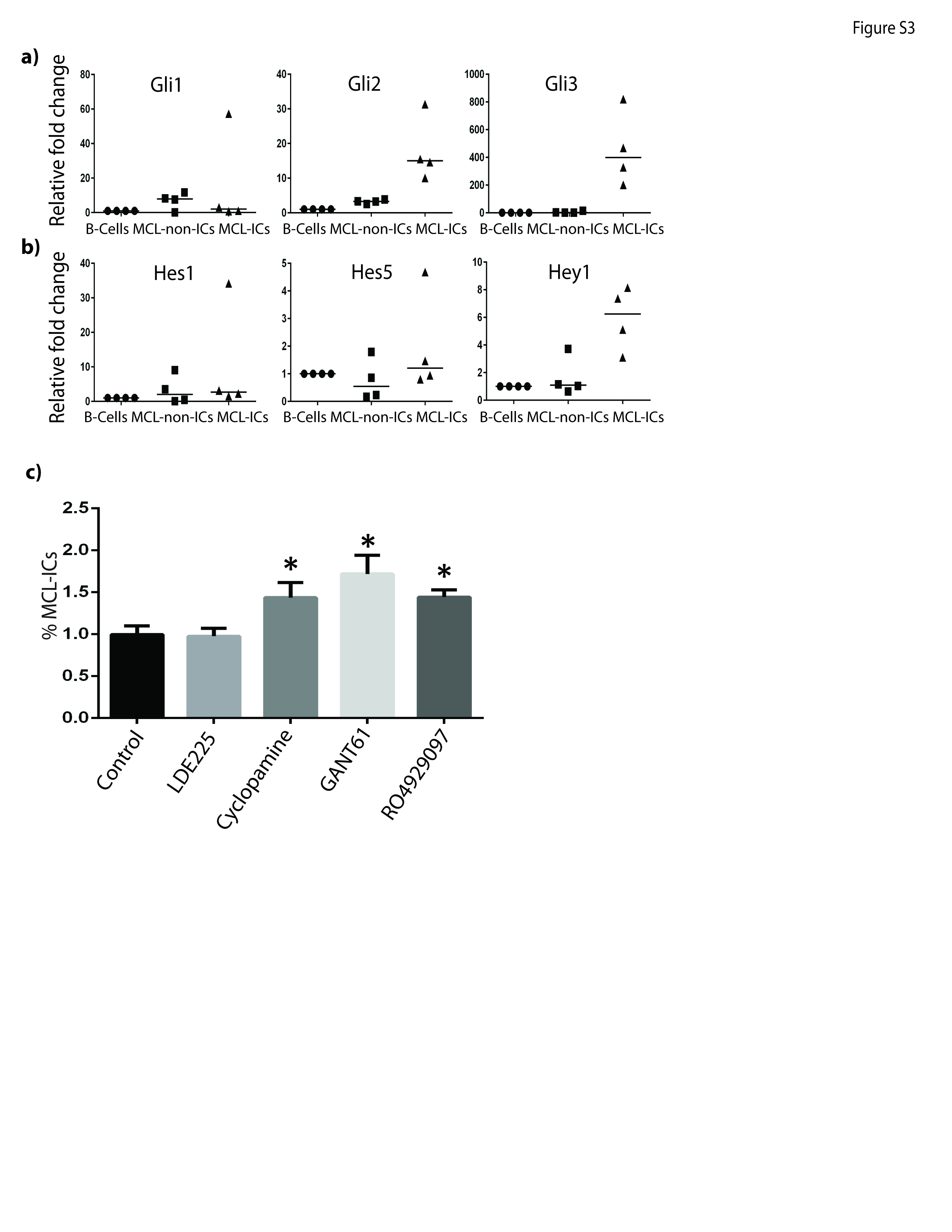

Supplement: Additional file 3: Figure S3. — Hedgehog and Notch signaling pathways in MCL-ICs. Expression of mRNA encoding (a) Hedgehog signaling pathway transcription factors Gli1, Gli2, Gli3 and (b) Notch signaling target genes Hes1, Hes5 and Hey1 in freshly isolated MCL-ICs, and MCL-non-ICs relative to B-cells from healthy donors. Horizontal lines represent median for each group. Differences between MCL-ICs and MCL-non-ICs were significant (P < 0.05) for Gli3, and Hey1. (c) Percentage of MCL-ICs evaluated by immunostaining and flow cytometry (as shown in Fig. 1a) of primary MCL cells (n = 3) treated with Hedgehog inhibitors LDE225 (5 μM), Cyclopamine (5 μM), GANT61 (5 μM) and Notch inhibitor RO4929097 (5 μM) for 48 h. *Differences between treated and control group were significant P < 0.05. Overexpression of Gli3, a repressor of hedgehog pathway [55, 56] in MCL-ICs and inability of hedgehog and notch signaling pathway inhibitors to decrease percentage of MCL-ICs, suggest that these pathways may not be effective targets for reducing the percentage of MCL-ICs. [file 13045_2015_161_MOESM3_ESM.tif]
